# Supplementary material for: Honey bee sting pain index by body location
Source: PeerJ. 2014 Apr 3;2:e338. doi: 10.7717/peerj.338 (PMC3994616; doi:10.7717/peerj.338)
Supplement: Supplemental Information [file peerj-02-338-s001.docx]

TITLE:

Honey Bee Sting Pain Index by Body Location

AUTHOR:

Michael L. Smith (corresponding author)

SUPPLEMENTARY INFORMATION:

Supplementary Data Table 1

Table S1:

Ratings for each individual sting location, including the side of the body stung, the date, the time, and the round. Ratings for the internal standard (forearm) were removed.

| Body Location | Side  (Left or Right) | Date | Time | 1^st^/2^nd^/3^rd^ Round | Rating |
| --- | --- | --- | --- | --- | --- |
| Abdomen | L | 27-Aug | 09:17 | 1st | 6 |
| Abdomen | R | 13-Sep | 09:09 | 2nd | 7 |
| Abdomen | R | 22-Sep | 09:09 | 3rd | 7 |
| Armpit | L | 30-Aug | 09:10 | 1st | 7 |
| Armpit | R | 11-Sep | 09:24 | 2nd | 7 |
| Armpit | L | 20-Sep | 09:09 | 3rd | 7 |
| Lower back | L | 22-Aug | 09:32 | 1st | 3 |
| Lower back | R | 10-Sep | 09:36 | 2nd | 3 |
| Lower back | L | 22-Sep | 09:16 | 3rd | 6 |
| Back of the neck | NA | 3-Sep | 09:31 | 1st | 6 |
| Back of the neck | NA | 11-Sep | 09:12 | 2nd | 6 |
| Back of the neck | NA | 24-Sep | 09:05 | 3rd | 4 |
| Behind the ear | L | 3-Sep | 09:23 | 1st | 6 |
| Behind the ear | R | 4-Sep | 09:17 | 2nd | 4 |
| Behind the ear | R | 17-Sep | 09:23 | 3rd | 6 |
| Buttock | L | 28-Aug | 09:05 | 1st | 3 |
| Buttock | R | 6-Sep | 09:15 | 2nd | 4 |
| Buttock | L | 25-Sep | 09:11 | 3rd | 4 |
| Calf | L | 21-Aug | 09:28 | 1st | 4 |
| Calf | R | 6-Sep | 09:22 | 2nd | 3 |
| Calf | R | 26-Sep | 09:16 | 3rd | 4 |
| Cheek | L | 21-Aug | 09:42 | 1st | 7 |
| Cheek | R | 12-Sep | 09:30 | 2nd | 7 |
| Cheek | L | 25-Sep | 09:18 | 3rd | 7 |
| Middle finger tip | R | 27-Aug | 09:24 | 1st | 7 |
| Middle finger tip | L | 13-Sep | 09:17 | 2nd | 7 |
| Middle finger tip | L | 17-Sep | 09:16 | 3rd | 6 |
| Foot arch | R | 3-Sep | 09:12 | 1st | 7 |
| Foot arch | L | 10-Sep | 09:30 | 2nd | 4 |
| Foot arch | R | 17-Sep | 09:29 | 3rd | 4 |
| Nostril | R | 28-Aug | 09:11 | 1st | 9 |
| Nostril | L | 13-Sep | 09:27 | 2nd | 9 |
| Nostril | R | 21-Sep | 09:25 | 3rd | 9 |
| Back of the knee | R | 20-Aug | 09:24 | 1st | 5 |
| Back of the knee | L | 4-Sep | 09:11 | 2nd | 6 |
| Back of the knee | R | 25-Sep | 09:06 | 3rd | 4 |
| Upper lip | R | 22-Aug | 09:20 | 1st | 9 |
| Upper lip | L | 10-Sep | 09:18 | 2nd | 9 |
| Upper lip | R | 26-Sep | 09:28 | 3rd | 8 |
| Nipple | R | 24-Aug | 09:21 | 1st | 7 |
| Nipple | L | 11-Sep | 09:18 | 2nd | 6 |
| Nipple | R | 24-Sep | 09:21 | 3rd | 7 |
| Palm | L | 20-Aug | 09:17 | 1st | 7 |
| Palm | R | 7-Sep | 09:17 | 2nd | 7 |
| Palm | L | 21-Sep | 09:39 | 3rd | 7 |
| Scrotum | NA | 24-Aug | 09:12 | 1st | 7 |
| Scrotum | NA | 5-Sep | 09:36 | 2nd | 7 |
| Scrotum | NA | 24-Sep | 09:11 | 3rd | 7 |
| Penis shaft | NA | 22-Aug | 09:07 | 1st | 7 |
| Penis shaft | NA | 7-Sep | 09:24 | 2nd | 8 |
| Penis shaft | NA | 19-Sep | 09:06 | 3rd | 7 |
| Skull | NA | 24-Aug | 09:37 | 1st | 3 |
| Skull | NA | 5-Sep | 09:31 | 2nd | 2 |
| Skull | NA | 19-Sep | 09:18 | 3rd | 2 |
| Middle toe tip | R | 20-Aug | 09:08 | 1st | 2 |
| Middle toe tip | L | 12-Sep | 09:13 | 2nd | 2 |
| Middle toe tip | R | 22-Sep | 09:22 | 3rd | 3 |
| Top of the foot | R | 30-Aug | 09:21 | 1st | 7 |
| Top of the foot | L | 7-Sep | 09:38 | 2nd | 6 |
| Top of the foot | R | 21-Sep | 09:19 | 3rd | 5 |
| Top of the hand | R | 21-Aug | 9:34 | 1st | 6 |
| Top of the hand | L | 5-Sep | 9:17 | 2nd | 6 |
| Top of the hand | R | 20-Sep | 9:22 | 3rd | 4 |
| Upper arm | R | 30-Aug | 9:16 | 1st | 2 |
| Upper arm | R | 4-Sep | 9:22 | 2nd | 3 |
| Upper arm | R | 20-Sep | 9:17 | 3rd | 2 |
| Upper thigh | L | 27-Aug | 9:37 | 1st | 4 |
| Upper thigh | R | 12-Sep | 9:21 | 2nd | 6 |
| Upper thigh | R | 19-Sep | 9:12 | 3rd | 4 |
| Wrist | R | 28-Aug | 9:23 | 1st | 4 |
| Wrist | L | 6-Sep | 9:07 | 2nd | 5 |
| Wrist | L | 26-Sep | 9:10 | 3rd | 5 |
